# Impact of Local Liver Irradiation Concurrent Versus Sequential with Lenvatinib on Pharmacokinetics and Biodistribution

**DOI:** 10.3390/cancers13071598

**Published:** 2021-03-30

**Authors:** Tung-Hu Tsai, Yu-Jen Chen, Li-Ying Wang, Chen-Hsi Hsieh

**Affiliations:** 1 Institute of Traditional Medicine, School of Medicine, National Yang Ming Chiao Tung University, Taipei 112, Taiwan; thtsai@ym.edu.tw (T.-H.T.); oncoman@mmh.org.tw (Y.-J.C.); 2 Departments of Radiation Oncology, Mackay Memorial Hospital, Taipei 104, Taiwan; 3 Department of Medical Research, China Medical University Hospital, Taichung 404, Taiwan; 4 Department of Nursing, MacKay Junior College of Medicine, Nursing and Management, Taipei 112, Taiwan; 5 School and Graduate Institute of Physical Therapy, College of Medicine, National Taiwan University, Taipei 100, Taiwan; liying@ntu.edu.tw; 6 Physical Therapy Center, National Taiwan University Hospital, Taipei 100, Taiwan; 7 Faculty of Medicine, School of Medicine, National Yang Ming Chiao Tung University, Taipei 112, Taiwan; 8 Division of Radiation Oncology, Department of Radiology, Far Eastern Memorial Hospital, New Taipei City 220, Taiwan

**Keywords:** biodistribution, hepatocellular carcinoma, lenvatinib, pharmacokinetics, radiotherapy

## Abstract

**Simple Summary:**

Lenvatinib is a systemic treatment for patients with advanced hepatocellular carcinoma (HCC). Stereotactic body radiation therapy (*SBRT*) is an advanced technique of hypofractionated external beam radiotherapy (EBRT) that can be applied in patients with HCC. The current study showed that the area under the concentration–time curve of lenvatinib concentration (AUC_lenvatinib_) increased by 148.8% with radiotherapy (RT)_2Gy×3f’x_ (EBRT for the whole liver), and 68.9% with RT_9Gy×3f’×_ (SBRT targeting a 1.5 × 1.5 cm region in the center of the liver) in the sequential regimen compared to the concurrent regimen in rats. Additionally, the AUC_lenvatinib_ was decreased by 50% in the concurrent regimen of both RT techniques with lenvatinib compared to the control group. The biodistribution of lenvatinib in the organs at risk was markedly decreased in the concurrent regimens. The radiation–drug interactions were between lenvatinib and RT, and showed sequential preferably.

**Abstract:**

Concurrent and sequential regimens involving radiotherapy (RT) and lenvatinib were designed with off-target or stereotactic body radiation therapy (SBRT) doses in a freely moving rat model to evaluate the effect of RT on the pharmacokinetics (PK) of lenvatinib. Liver RT concurrent with lenvatinib decreased the area under the concentration–time curve of lenvatinib concentration (AUC_lenvatinib_) by 51.1% with three fractions of 2 Gy (RT_2Gy×3f’x_, *p* = 0.03), and 48.9% with RT_9Gy×3f’x_ (*p* = 0.03). The AUC_lenvatinib_ increased by 148.8% (*p* = 0.008) with RT_2Gy×3f’x_, and 68.9% (*p* = 0.009) with RT_9Gy×3f’x_ in the sequential regimen compared to the concurrent regimen. There were no differences in the AUC_lenvatinib_ between RT_2Gy×3f’x_ and RT_9Gy×3f’x_ in the concurrent or sequential regimen. Both the RT_2Gy×3f’x_ and RT_9Gy×3f’x_ concurrent regimens markedly decreased the biodistribution of lenvatinib in the heart, liver, lung, spleen, and kidneys, which ranged from 31% to 100% for RT_2Gy×3f’x_, and 11% to 100% for RT_9Gy×3f’x_, compared to the sham regimen. The PK and biodistribution of lenvatinib can be modulated by simultaneous off-target irradiation and SBRT doses. The timing of lenvatinib administration with respect to RT, impacted the PK and biodistribution of the drug. Additionally, off-target and SBRT doses had a similar ability to modulate the effect of systemic therapy.

## 1. Introduction

The incidence of hepatocellular carcinoma (HCC) and the associated mortality are increasing worldwide [1]. The oral multikinase inhibitor sorafenib (Nexavar, Bayer Pharma AG, Berlin, Germany) provides a clinically significant improvement in the overall survival of HCC patients [2,3]. Recently, the phase III REFLECT trial showed that lenvatinib (Eisai Inc., Woodcliff Lake, NJ, USA) was noninferior to sorafenib for the overall survival of patients with HCC [4]. Therefore, the National Comprehensive Cancer Network^®^ Clinical Practice Guidelines in Oncology list both lenvatinib and sorafenib as first-line systemic treatments for HCC patients.

Stereotactic body radiation therapy (SBRT) is an advanced external beam radiotherapy (EBRT) technique, which can be considered as an alternative to ablation/embolization techniques, or used when these techniques fail or are contraindicated for HCC [5,6]. Recently, impressive benefits have been reported for radiotherapy (RT) combined with sorafenib in patients with unresectable HCC [7,8], but severe adverse effects have also been reported [9,10]. Moreover, interactions between RT and sorafenib, regardless of the target or off-target radiation dose, were confirmed by pharmacokinetics (PK) in a freely moving rat model [8].

Nuclear factor kappa B (NF-κB) modulates the expression of cytochrome P450 (CYP) 3A4 [11], increasing the expression of platelet-derived growth factor (PDGF) and vascular endothelial growth factor (VEGF) [12]. NF-κB responds to environmental stress, including RT [13]. Additionally, the expression of CYP3A4 can be affected by RT [8]. Furthermore, lenvatinib targets: VEGFR1, 2, and 3; fibroblast growth factor receptor (FGFR) 1, 2, 3, and 4; PDGFR-alpha (α); the RET proto-oncogene; and c-kit [14,15], and is metabolized primarily in the liver, undergoing oxidative metabolism via CYP3A4 [16].

Taken together, these data suggest that there may be interactions between RT and lenvatinib. In the current study, the PK behavior of lenvatinib with different RT doses and schedules was evaluated. Furthermore, the biodistribution of lenvatinib with, and without, RT was evaluated to provide suggestions for clinical applications.

## 2. Materials and methods

### 2.1. Chemicals and Reagents

Lenvatinib and cyclosporin A were purchased from Sigma-Aldrich (St. Louis, MO, USA). As an internal standard, biochanin A was purchased from Toronto Research Chemicals Inc. (North York, ON, Canada). Polyethylene glycol 400 (PEG 400) and heparin sodium were purchased from Sigma-Aldrich. Pentobarbital sodium was obtained from SCI Pharmtech (Taoyuan, Taiwan). The solvents and reagents for chromatography were purchased from J.T. Baker (Phillipsburg, NJ, USA) and Merck (Darmstadt, Germany). Standard solutions of lenvatinib and biochanin A in methanol were stored at −20 °C. Triply deionized water from Millipore (Bedford, MA, USA) was used for all preparations.

### 2.2. High-Performance Liquid Chromatography-Ultraviolet (HPLC-UV)

**Figure 1:**
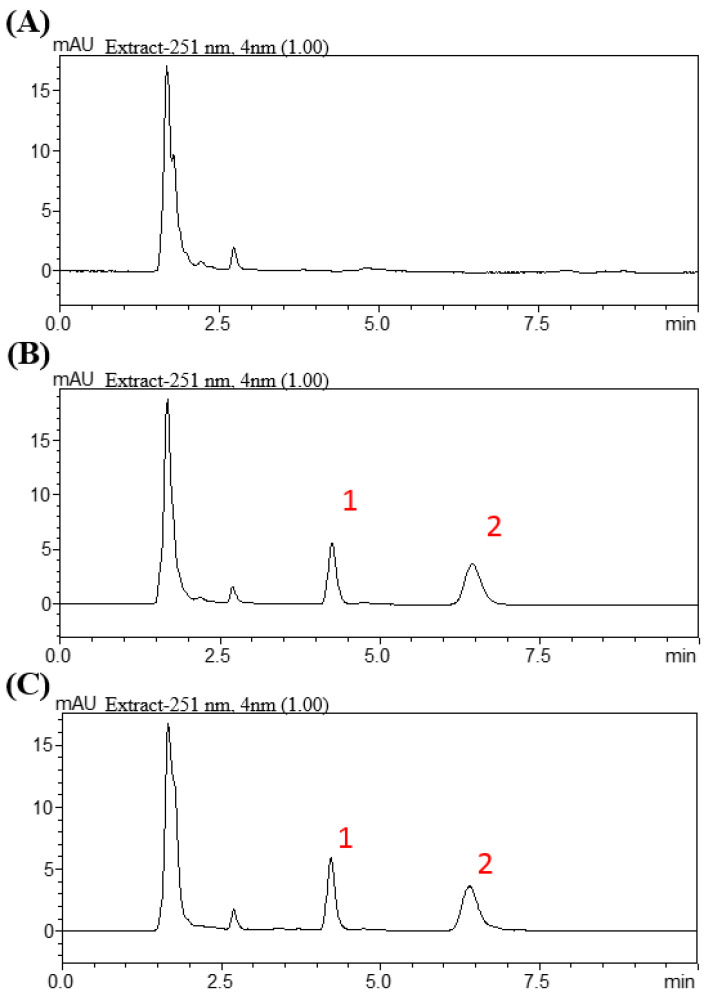
Chromatograms resulting from high-performance liquid chromatography for lenvatinib. (**A**) Blank. (**B**) Blank spiked with lenvatinib (1 µg/mL) and biochanin A (0.75 µg/mL). (**C**) Plasma Scheme 60 min after lenvatinib administration (3 mg/kg, p.o.). Peak 1: biochanin A (0.75 µg/mL). Peak 2: lenvatinib (0.94 µg/mL).

The HPLC system consisted of a chromatographic pump (LC−20AT), an online injector (SIL−20C) equipped with a 10 μL sample loop to inject the sample, and an ultraviolet detector (SPD-M20A). Lenvatinib and samples were separated on an Agilent ZORBAX SB-phenyl column (Agilent Technologies, Santa Clara, CA, USA, 150 × 4.6 mm i.d., particle size = 5 μm). The mobile phase for the lenvatinib group consisted of water and acetonitrile (30:70, *v:v*) at a flow rate of 1 mL/min. A wavelength of 251 nm was set for the optimal photodiode-array detection of lenvatinib. Lenvatinib had a retention time of 6.5 min, with good separation and no endogenous interference in the rat plasma samples, and the procedure exhibited good selectivity (Figure 1).

### 2.3. Method Validation: Calibration Curve

The calibration curves covered a concentration range from 0.1 to 50 μg/mL. The linearity of the assay was determined using the coefficient of determination (*r*^2^) for the calibration curve, which should be greater than 0.995. The limit of detection (LOD) was defined as the concentration that generated a signal-to-noise ratio of 3, and the lower limit of quantification (LLOQ) was defined as the lowest concentration within the linear regression that yielded a signal-to-noise ratio of 10. A 0.01 mg/mL limit of quantification was identified as the lowest concentration on the calibration curve that could be measured routinely with acceptable bias and relative SD. Calibration standards for plasma samples were prepared by adding known amounts of lenvatinib (10 μL) into rat plasma blanks (40 μL each) to yield a range of 0.05–50 μg/mL. These mixtures were supplemented with 150 μL of internal standard solution (10 μg/mL).

### 2.4. Method Validation: Precision, Accuracy, and Recovery

The intra- and interassay variability for lenvatinib was determined by quantifying six replicates at concentrations of 0.05, 0.1, 0.5, 1, 5, and 10 μg/mL, using the above-described HPLC method on the same day, and on six consecutive days. The accuracy (% bias) was calculated from the nominal concentration (*C*_nom_) and the mean value of observed concentrations (*C*_obs_) as follows: accuracy (% bias) = ((*C*_nom_ − *C*_obs_)/*C*_nom_) × 100. The relative standard deviation (RSD) was calculated from the observed concentrations as follows: precision (% RSD) = (standard deviation (SD)/*C*_obs_) × 100. The same data were used to determine both accuracy and precision. The relative error and coefficient of variation were maintained within ± 15%, except for the LLOQ, which was not permitted to exceed ± 20%. Recovery was assessed at three different concentrations (0.05, 0.5 and 10 μg/mL) by comparing the peak areas of the post-extraction spiked samples with those of the standard solution.

### 2.5. Experimental Animals and Drug Administration

#### 2.5.1. Animals and Sample Preparation

The study protocol was reviewed and approved by the Institutional Animal Experimentation Committee of National Yang-Ming University, Taipei, Taiwan, and by the Institutional Animal Care and Use Committee (IACUC, approval number 1080314). Adult male Sprague-Dawley rats (300 ± 20 g body weight) were provided by the Laboratory Animal Center at National Yang-Ming University (Taipei, Taiwan). The animals had access to water and food ad libitum (laboratory rodent diet 5P14, PMI Feeds, Richmond, IN, USA) and lived in a pathogen-free environment, with a 12/12 h light–dark cycle. All animal experiments followed the guidelines and procedures for the care of laboratory animals at National Yang-Ming University.

#### 2.5.2. Irradiation Technique

A freely moving rat model was designed for the current study [8]. The rats were anesthetized and immobilized on a board while undergoing computed tomography to localize the whole liver or a central area measuring 1.5 cm × 1.5 cm for the SBRT technique. For the whole liver field, the cranial margin was set 5 mm from the top of the diaphragm, and the caudal margin was set 5 mm lower than the liver margin. The whole liver was targeted for irradiation. The experimental animals were randomized into groups receiving three fractions of sham RT, RT_2Gy_, or RT_9Gy_, given concurrently or sequentially with lenvatinib. Data were collected from six rats per group.

#### 2.5.3. Drug Delivery with Different Schedules and Doses of RT

**Figure 2:**
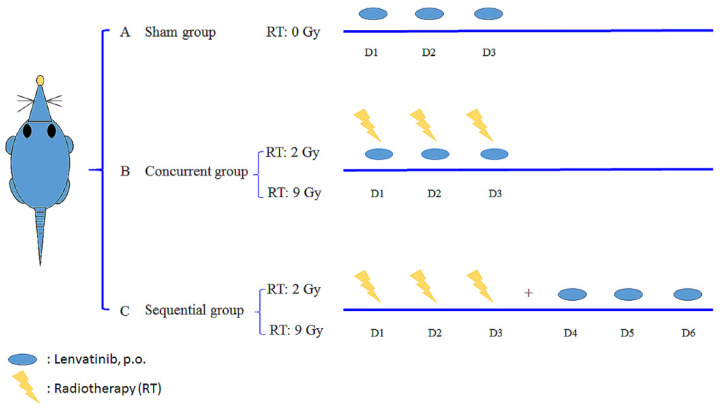
Oral lenvatinib [3 mg/kg per os (p.o.), quaque die (q.d.)] with radiotherapy (RT) under different time schedules and RT doses. The rats were randomly divided into a sham group, lenvatinib (p.o., q.d. × 3 d) with RT_0Gy_ (lenvatinib_×3d_); a concurrent group, lenvatinib_×3d_ 1 h after RT_2Gy_ in 3 fractions (RT_2Gy×3f’x_) and RT_9Gy×3f’x_; and a sequential group, lenvatinib_×3d_ 24 h after RT_2Gy×3f’x_ and RT_9Gy×3f’x_. Data are expressed as the mean ± standard error of the mean.

A radiation dose of 2 Gy was considered the daily conventional EBRT dose or the off-target dose around the target that received an ablation RT dose. A dose of 9 Gy was used to simulate SBRT. The animals were divided into five groups, as follows: (A) sham group, lenvatinib (3 mg/kg) only for 3 days (d) with RT_0Gy_ (lenvatinib_×3d_); (B) whole-liver (2 Gy, 3 fractions) RT_2Gy×3f’x_ concurrent with 3 d of lenvatinib (3 mg/kg); (C) whole-liver RT_2Gy×3f’x_ followed by 3 d of lenvatinib (3 mg/kg); (D) SBRT (9 Gy with 3 fractions) RT_9Gy×3f’x_ concurrent with lenvatinib (3 mg/kg) for 3 d; and (E) RT_9Gy×3f’x_ followed by 3 days of lenvatinib (3 mg/kg) for 3 d (Figure 2). Rats were initially anesthetized with pentobarbital (50 mg/kg, i.p.) and remained anesthetized throughout the experimental period. After surgery, the rats were placed in an experimental cage and allowed to recover for 1 day. Lenvatinib was dissolved in triply deionized water and administered [3 mg/kg, per os (p.o.)] to the rats (*n* = 6 per group).

#### 2.5.4. Pretreatment with Ketoconazole for Drug Delivery with RT under Different Time Schedules and Doses

The effects of ketoconazole (KZT), an inhibitor of CYP3A [17] and P-gp [18], were investigated in rats treated with lenvatinib. The rats were pretreated with KZT (10 mg/kg, i.p.) 30 min before RT or lenvatinib. After 30 min, lenvatinib (3 mg/kg) was administered intragastrically. Blood samples were obtained according to a preset schedule.

#### 2.5.5. Sample Preparation

Blood samples were collected in a heparin-rinsed vial via polyethylene tubing (PE-50) implanted into the jugular vein of each rat. Aliquots of 100–120 µL blood were collected at time intervals of 0, 5, 15, 30, 45, 60, 90, 120, 150, 180, 210, and 240 min following drug administration. At each time point, 200 μL of blood was drawn into heparin-rinsed Eppendorf tubes and then centrifuged at 13,000 rpm for 10 min at 4 °C to obtain plasma. Plasma was stored at −20 °C until analysis. Each collected blood sample was transferred to a heparinized microcentrifuge tube and centrifuged at 13,000 rpm for 10 min. The resulting plasma (50 μL) was then mixed with 150 μL of internal standard solution (10 μg/mL). The denatured protein precipitate was separated by vortexing for 20 s and centrifuged at 13,000 rpm for 10 min at 4 °C.

#### 2.5.6. Organ Distribution

Six hours after lenvatinib administration_×3d_ (3 mg/kg, p.o.), blood samples were collected as mentioned above. The brain, liver, heart, spleen, lung, and kidney were collected and weighed. These collected samples were stored at −20 °C until analysis.

#### 2.5.7. Organ Samples

The thawed organ samples were homogenized in 50% aqueous acetonitrile (the ratio of sample weight and volume was 1:5), and the homogenate was centrifuged at 13,000× *g* for 10 min at 4 °C. The supernatant was collected, placed in brown Eppendorf tubes, and stored at −20 °C until analysis. Briefly, each organ sample (50 μL) was combined with 150 μL of IS solution (diethylstilbestrol) for protein precipitation. Finally, 20 μL of filtrate was subjected to HPLC analysis.

#### 2.5.8. Hepatic and Renal Functions

Glutamic-pyruvic transaminase (GPT) and creatine were measured to examine the influence of different modalities on hepatic function and renal function by a standard colorimetric method using a Synchron L × 20 spectrophotometer (Beckman Coulter, Lakeview Pkwy S Dr, IN, USA) and manufacturer-supplied reagents.

### 2.6. Pharmacokinetics and Data Analysis

PK parameters, including the area under the concentration–time curve (AUC), clearance (CL), elimination half-life (*t*_1/2_), volume of distribution at steady state (Vss), and mean residence time (MRT), were calculated using the PK calculation software WinNonlin Standard Edition, Version 1.1 (Scientific Consulting, Apex, NC, USA) by a compartmental method.

### 2.7. Calculations and Data Analysis

All statistical calculations were performed with Statistical Product and Service Solutions (SPSS) for Windows, version 20.0 (SPSS, IBM, Armonk, NY, USA). All data are expressed as the mean ±SD. One-way analysis of variance (ANOVA) was used for comparisons between groups, and statistically significant differences were defined as * *p* < 0.05 or ** *p* < 0.01.

## 3. Results

### 3.1. Method of Validation for Linearity, Recovery, Precision, Accuracy, and Stability

In the current study, the LOD of lenvatinib in plasma was 0.5 μg/mL. The regression equation for lenvatinib in rat plasma was *y* = 1.6777 × −0.0751 (r^2^ = 1). The intraday accuracy (% bias) of lenvatinib ranged from 2.39 to 23.3%. The intraday precision (% RSD) ranged from 2.35 to 23.8%. The recovery rate of lenvatinib at 0.05 to 10 µg/mL ranged from 111.8% to 96.15%.

### 3.2. Both RT_2Gy_ and RT_9Gy_ Modulated the AUC of Lenvatinib in the Plasma of Freely Moving Rats

**Figure 3:**
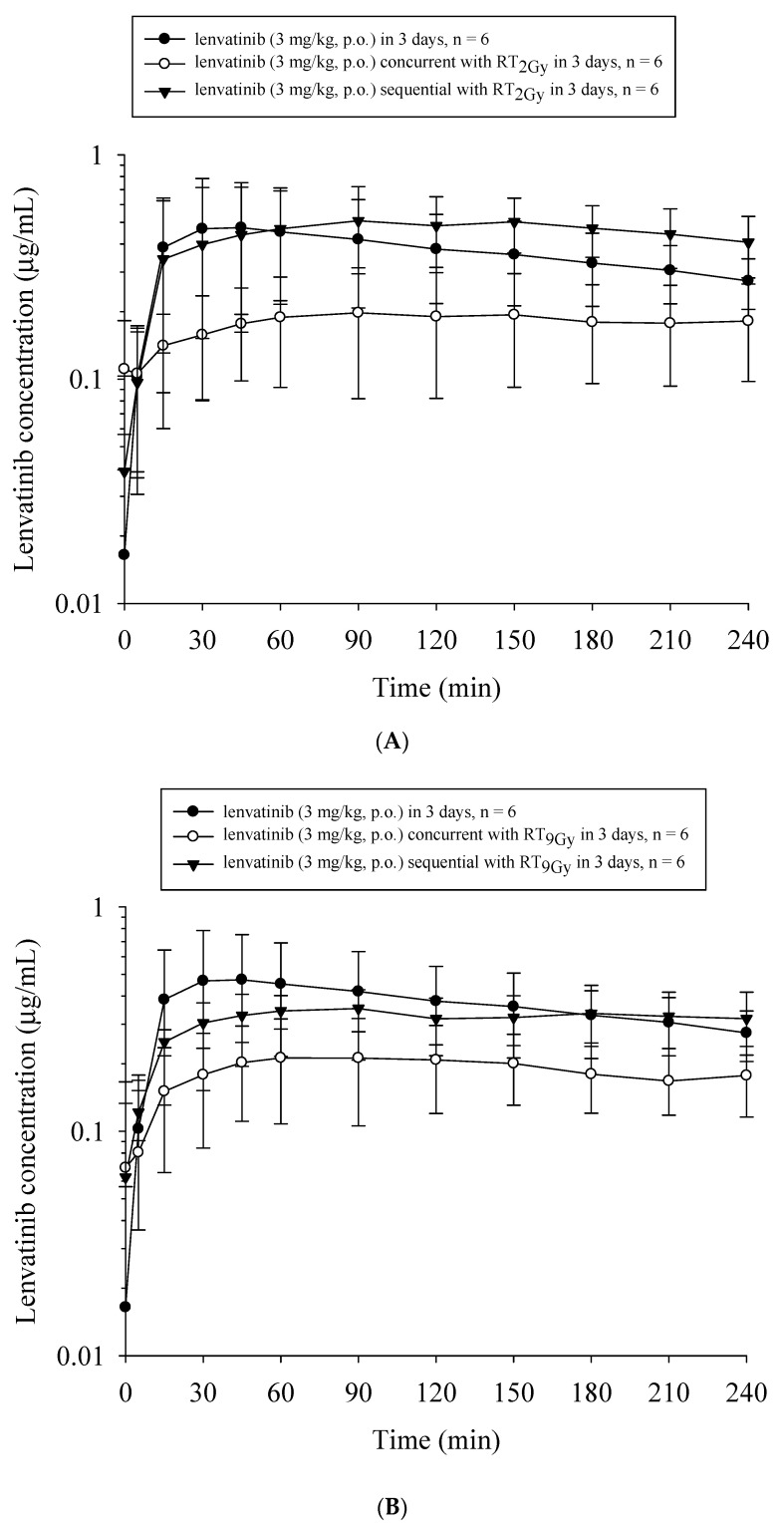
The concentration–time curves of lenvatinib in the plasma of rats under different treatment schedules with or without radiotherapy (RT). The treated groups in (**A**) included the sham group, which received lenvatinib (p.o., q.d._×3d_) with RT_0 Gy_ (lenvatinib_×3d_); the concurrent group, which received lenvatinib_×3d_ 1 h after 3 fractions of RT_2Gy_ (RT_2Gy×3f’x_); and the sequential group, which received lenvatinib_×3d_ 24 h after RT_2Gy×3f’x_. The treated groups in (**B**) included the sham group, which received lenvatinib_×3d_; the concurrent group, which received lenvatinib_×3d_ 1 h after RT_9 Gy_ in 3 fractions (RT_9Gy×3f’x_); and the sequential group, which received lenvatinib_×3d_ 24 h after RT_9Gy×3f’x_. Data are expressed as the mean ± standard error of the mean (*n* = 6 per group).

Compared to lenvatinib_×3d_, the AUC_lenvatinib_ and C_max_ decreased by 51.1% (*p* = 0.03) and 58.3% (*p* = 0.03), respectively, with RT_2Gy×3f’x_ in the concurrent regimen. However, the AUC_lenvatinib_ increased by 21.6% with RT_2Gy×3f’x_ in the sequential regimen (*p* = 0.44). Additionally, compared to the concurrent regimen, the AUC_lenvatinib_ and C_max_ increased by 148.8% (*p* = 0.008) and 154.5% (*p* = 0.008), respectively, in the sequential RT_2Gy×3f’x_ regimen. Moreover, the AUC_lenvatinib_ did not differ between the concurrent RT_2Gy×3f’x_ and RT_9Gy×3f’x_ regimens (*p* = 0.87). (Figure 3A)

Similarly, the AUC_lenvatinib_ and C_max_ decreased by 48.9% (*p* = 0.03) and 56.1% (*p* = 0.04), respectively, with the concurrent RT_9Gy×3f’x_ regimen. There was no statistically significant difference in the AUC_lenvatinib_ between the lenvatinib _x 3d_ and sequential RT_9Gy×3f’x_ groups. Intriguingly, the AUC_lenvatinib_ and C_max_ increased by 68.9% (*p* = 0.009) and 68.7% (*p* = 0.02), respectively, with the sequential RT_9Gy×3f’x_ group compared to the concurrent regimen. Furthermore, there were no statistically significant differences in the AUC_lenvatinib_ between the sequential RT_2Gy×3f’x_ and RT_9Gy×3f’x_ groups (*p* = 0.12). (Figure 3B)

**Figure 4:**
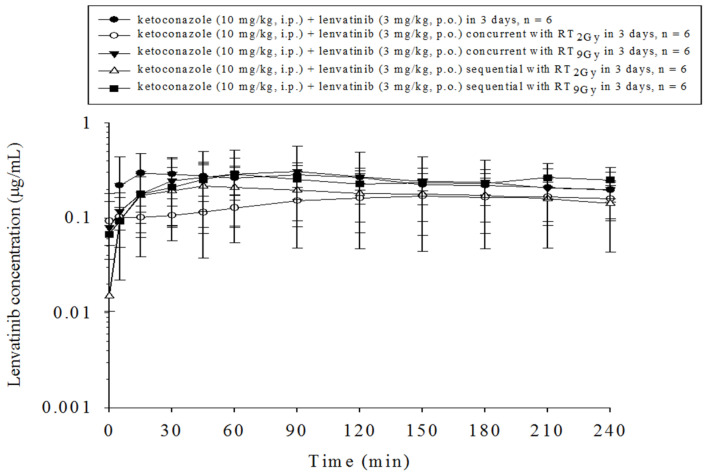
The concentration–time curves of lenvatinib in the plasma of rats that received lenvatinib coadministered with ketoconazole with or without radiotherapy (RT) under different time courses. Data are expressed as the mean ± standard error of the mean (*n* = 6 per group).

Compared to RT concurrent or sequential with lenvatinib, the coadministration of ketoconazole with RT and lenvatinib mildly decreased the AUC_lenvatinib_ in all groups except for the concurrent RT_9 Gy_ group, which showed a nonsignificant 28% increase (Figure 4 and Table 1).

### 3.3. Organ Distribution under Different RT and Lenvatinib Regimens

**Figure 5:**
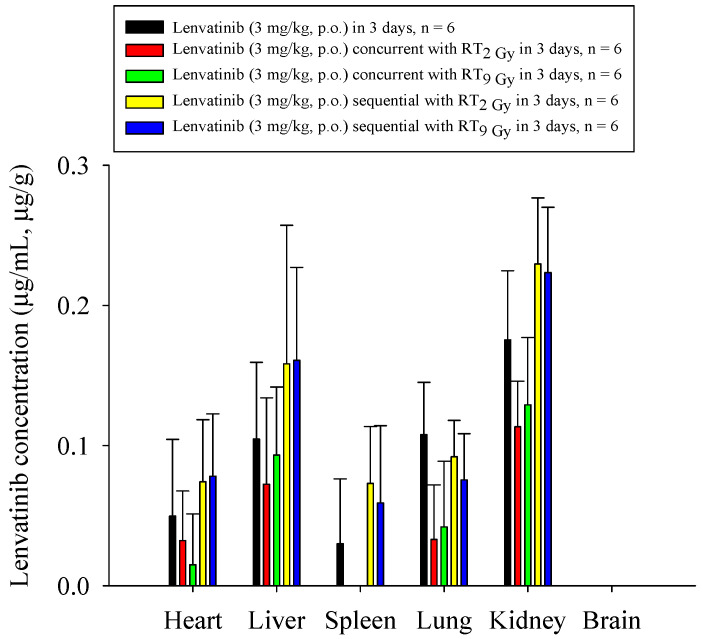
The concentration of lenvatinib in the heart, liver, spleen, lung, kidney, and brain of rats after the administration of lenvatinib (3 mg/kg, p.o.) with or without radiotherapy. The lenvatinib concentrations in the organs are expressed in units of µg/mL or µg/g (*n* = 6 per group).

Both the RT_2Gy×3f’x_ and RT_9Gy×3f’x_ concurrent regimens markedly decreased the biodistribution of lenvatinib in the heart, liver, lung, spleen, and kidneys, which ranged from 31% to 100% for RT_2Gy×3f’x_, and 11% to 100% for RT_9Gy×3f’x_, compared to the sham regimen. In contrast, the RT_2Gy×3f’x_ and RT_9Gy×3f’x_ sequential regimens increased the biodistribution of lenvatinib in the heart, liver, spleen, and kidneys, which ranged from 31% to 143% for RT_2Gy×3f’x_ and 27% to 100% for RT_9Gy×3f’x_, compared to the control regimen. However, the concentration of lenvatinib in the lung in the RT_2Gy×3f’x_ and RT_9Gy×3f’x_ sequential regimens was decreased by 15% and 31%, respectively, although the differences were not statistically significant. Intriguingly, lenvatinib was not detected in the brain. Additionally, the concentration of lenvatinib in the spleen increased more than 100% during the sequential regimen (Figure 5 and Table 2).

## 4. Discussion

To our knowledge, our study is the first to show the interaction between RT and lenvatinib. The AUC of lenvatinib could be modulated by RT with off-target and SBRT doses. Additionally, RT concurrent or sequential with lenvatinib impacted the PK and biodistribution. Together, these data support the RT–PK phenomenon in our study.

Sorafenib is a multikinase inhibitor that targets the Raf/MAPK/ERK signaling pathway, interfering with the tyrosine kinases VEGFR-2/3, PDGFR-β, B-Raf, c-Raf, FGFR1, Flt3, c-KIT, RET, and p38 α, and induces tumor cell apoptosis in HCC [19,20]. Clinically, sorafenib provides overall survival benefits for patients with unresectable HCC, as was confirmed in the Sorafenib HCC Assessment Randomized Protocol (SHARP) [2] and Asia-Pacific [3] trials. Moreover, preclinical data from studies combining sorafenib and RT have suggested improved efficacy in HCC cell lines both in vitro and in vivo [21,22]. Additionally, the efficacy of RT with sorafenib in patients with unresectable HCC has been reported [7,8]. Moreover, the AUC of sorafenib can be modulated by RT [8].

Lenvatinib is an anticancer drug for the treatment of thyroid cancer [23], renal cancer [24], and HCC [4]. Lenvatinib concurrently targets: VEGFR1, 2, and 3; FGFR1, 2, 3 and 4; PDGFR-α; the RET proto-oncogene; and c-kit [14,15]. Due to these properties, lenvatinib is a candidate tumor inhibitor, acting in the same manner as sorafenib. Moreover, the REFLECT trial showed a noninferior median survival time for HCC patients treated with lenvatinib compared to sorafenib [4]. Therefore, the National Comprehensive Cancer Network Guidelines^®^ list lenvatinib alongside sorafenib as a first-line treatment for patients with HCC.

Angiogenesis is initiated under inflammatory or hypoxic conditions [25]. VEGF is a cytokine that is associated with the formation of new blood vessels for tumors, increased tumor proliferation, and the growth of tumor cells [26]. Irradiation induces hypoxia and VEGF upregulation [27], as well as VEGFR2 receptors in the tumor endothelium [28], PDGF in endothelial cells [29], and PDGFR in fibroblasts [30]. Additionally, NF-κB responds to irradiation [31] and increases the expression of VEGF [12]. Agents that inhibit VEGF signaling can: “normalize” tumor blood vessels; create a radiosensitive microenvironment [27,32,33]; enhance radiation damage to endothelial cells by promoting vessel regression and tumor cell death; suppress waves of reoxygenation, and hence reduce radiotherapy resistance [34]; promote ceramide-mediated apoptosis, to enhance the effect of radiotherapy [35]; and reduce the acute mobilization of circulating endothelial cells and endothelial progenitor cells [27,32,33]. These data suggest that RT in combination with both VEGF and PDGF signaling inhibitors greatly enhances antiangiogenic and antitumor effects.

Moreover, RT induces inflammation and results in the recruitment of neutrophils, macrophages, plasma cells, and granulocytes to the target area [36,37,38]. Polymorphonuclear neutrophils (PMNs) promote the secretion of matrix metalloproteinase (MMP)−8, a member of the MMP family, when they are localized in inflammatory areas [39]. Collagenases (MMP−1, 8, and 13) are proteins associated with angiogenesis [40]. Additionally, MMP−8 secreted by irradiated nonparenchymal cells enhances the migration and invasion of HCC [41]. More importantly, MMP−8 has been demonstrated to play a major role in local RT-induced modulation of systemic PK of 5-fluorouracil (5-FU) [42]. It is reasonable to suspect that the inflammatory process induced by RT and the autocrine and paracrine functions of the VEGF and MMP families may jointly contribute to the RT-PK phenomenon.

Sorafenib [22] and regorafenib [43] selectively inhibit the radiation-induced activation of VEGFR and enhance the effectiveness of irradiation. Intriguingly, lenvatinib targets VEGFR and PDGFR, and significantly inhibits thyroid cancer growth when combined with RT [44]. Moreover, RT followed by sorafenib was associated with the greatest tumor growth delay, as reported by Plastaras et al. [45]. Additionally, the AUC of sorafenib can be upregulated by RT [8]. Interestingly, it has recently been reported that the AUC of regorafenib can be modulated by RT [46]. In other words, lenvatinib is a potential candidate for radiosensitization, and the PK of lenvatinib may be modulated by RT.

Both off-target and treatment doses have similar interactions with chemotherapy drugs, such as 5-FU [47,48] and cisplatin [49,50], and multikinase inhibitors, such as sorafenib [8]. Similarly, the current study also confirmed that off-target and SBRT doses were comparable in their ability to modulate the PK of lenvatinib. The AUC_lenvatinib_ decreased by approximately 50% with concurrent RT_2Gy×3f’x_ and RT_9Gy×3f’x_. However, the AUC_lenvatinib_ increased by 149% with RT_2Gy×3f’x_, and by 69% with RT_9Gy×3f’x_ in the sequential regimen compared to the concurrent regimen.

Similarly, the C_max_ of lenvatinib decreased by 58% with RT_2Gy×3f’x_, and by 56% with RT_9Gy×3f’x_ in the concurrent regimen. A decreased C_max_ during the concurrent regimen indicates that both off-target and SBRT doses in local liver RT reduce the effect on the absorption of lenvatinib. In other words, the sequential regimen has a greater ability than the concurrent regimen to maintain the absorption of lenvatinib. The REFLECT trial noted improvements in progression-free survival, time to progression, and objective response with lenvatinib compared to sorafenib [4]. Accordingly, the concentration of lenvatinib in the liver was increased by 50% with the sequential RT regimen in the current study. RT was found to be at its most effective when administered during the “normalization window” of angiogenesis inhibitors [51], suggesting that the scheduling of the two modalities impacts the maximum clinical benefit. These results suggest that the combination of RT and lenvatinib affects the PK of lenvatinib in the plasma, and that sequential regimens may be more impactful than concurrent regimens.

Lenvatinib is a substrate for P-glycoprotein, and the oxidative metabolism of lenvatinib is mediated primarily by CYP3A4. However, recent results have shown no clinically important alterations in lenvatinib exposure following the coadministration of lenvatinib with rifampin [16] or ketoconazole [52]. Similarly, our study also noted minimal changes in systemic lenvatinib exposure following the coadministration of lenvatinib and RT with ketoconazole. In other words, oxidative metabolism does not appear to be a major pathway involved in the action of lenvatinib or its coadministration with RT. It is possible that multiple pathways are involved in lenvatinib metabolism, and the coadministration of lenvatinib with CYP3A4 inhibitors during RT is not likely to result in clinically important alterations in lenvatinib exposure.

Patients with HCC treated by SBRT have achieved encouraging outcomes [5,6]. SBRT delivers highly conformal dose distributions, allowing dose escalation, but this is at the expense of a larger volume around the target receiving low to moderate doses compared to three-dimensional RT [53]. Blettner et al. [54] reported a significant nonlinear dose–response relationship compatible with a cell-killing effect at high doses. Coppes et al. [55] reported that the out-of-field effect of radiation was similar to the in-field effect. Moreover, the AUCs of chemotherapy drugs and multikinase inhibitors can be modulated by low-dose RT in free-moving rat models [8,42,50]. Interestingly, there were no differences in the AUC_lenvatinib_ between the concurrent RT_2Gy×3f’x_ and RT_9Gy×3f’x_ groups. Similarly, there were no differences in the AUC_lenvatinib_ between the sequential RT_2Gy×3f’x_ and RT_9Gy×3f’x_ groups. These observations suggest the parallel impact of off-target and treatment irradiation doses, especially in the same regimen. For this reason, low-dose “bath” effects should be considered in advanced radiotherapy techniques concurrent or sequential with lenvatinib.

Organ distributions decreased in the concurrent regimen and increased in the sequential regimen. In the sequential regimen, RT increased the concentration of lenvatinib by 50% in the heart and 30% in the kidneys. However, in the Phase III Study of (E7080) Lenvatinib in Differentiated Cancer of the Thyroid (SELECT), patients treated with lenvatinib experienced cardiac dysfunction (7%), nausea and vomiting (20–40%), stomatitis (20–30%), diarrhea (60%), fatigue (more than 50%), palmar-plantar erythrodysesthesia syndrome (70%), hemorrhagic events (35%), thrombocytopenia (8.8%), fistula formation (1.5%), reversible posterior leukoencephalopathy syndrome (0.4%), and thromboembolic events (5.4%), and one-third of patients experienced proteinuria of any grade [23,56]. Additionally, increased exposure to lenvatinib is correlated with an increased incidence and severity of treatment-emergent hypertension [57], which is a demonstrated class effect of agents targeting the VEGF/VEGFR signaling pathway [58]. Avoiding potential side effects through close follow-up is as important as pursuing better treatment effects with combined modalities. Intriguingly, lenvatinib was not detected in the brain. Lenvatinib is a substrate for P-glycoprotein [16], an efflux pump on the endothelial cells that comprise the blood–brain barrier (BBB), suggesting that lenvatinib may be unable to penetrate the BBB. In the development of new radiation-modulated strategies and the design of clinical trials, these unexpected biological enhancements of lenvatinib in the RT-PK phenomenon should be addressed cautiously, to avoid severe toxicity when RT and lenvatinib are used as synergistic tools in cancer treatment strategies.

There were some limitations to our study. First, the current study did not use orthotopic or heterotopic models, but used a freely moving Sprague-Dawley rat model to confirm the interaction between RT and lenvatinib. However, the differences in the PK profile of lenvatinib between healthy subjects and patients are small [59]. Therefore, it appears reasonable to use a nontumor model to evaluate the interaction between RT and lenvatinib.

Second, the current study did not include a disease model treated with lenvatinib and RT; therefore, we cannot report the treatment effects of the combination of RT and lenvatinib. However, the current analysis sheds light on the discrepancies of PK in the concurrent and sequential regimens of RT with lenvatinib, which may be useful for prospective clinical trial designs. Brade et al. [9] noted that concurrent SBRT and sorafenib may cause unpredictable toxicity. Our previous study also confirmed that the AUC of sorafenib was increased in concurrent regimens with SBRT and EBRT [8]. These data provide a preclinical proof of concept for the effects of RT impact multikinase inhibitor activity and the RT-PK phenomenon to support clinical practice, as well as the design and conduct of early-phase radiotherapy trials with targeted therapeutics.

Third, the possible mechanism was not examined in the current study, because the presence or absence of the RT-PK phenomenon in the context of lenvatinib plus RT could not be ensured before the study. However, we confirmed that the systemic PK of lenvatinib could be modulated by conventional EBRT and SBRT, as well as concurrent RT and sequential RT. The design of these combination regimens provides different scenarios in clinical practice. We realized that an in vitro study could not replicate or validate an in vivo study because the impacts of the local microenvironment and systemic modulators would be absent. Notably, the RT regimen affects P-gp activity, and the sequential RT regimen increases CYP3A4 activity to modulate the PK of sorafenib [8]. Therefore, there is a clear need for further in vitro studies to explore the mechanism of the RT-PK phenomenon of lenvatinib in the future; for example, by comparing various HCC tumor cell lines with nontumorigenic hepatic cells to assess cell viability, colony formation, cell cycle situations, DNA double-strand break, DNA repair, CYP 3A4 activity, and P-gp activity.

## 5. Conclusions

Taken together, these data support the concept of the interplay between RT and lenvatinib by suggesting that the systemic PK of lenvatinib can be modulated by irradiation. The timing of lenvatinib and RT doses affected the AUC and biodistribution of the drug during treatment. The AUC_lenvatinib_ was decreased by 50% in multiple fractions of RT concurrent with lenvatinib. Additionally, off-target and SBRT doses had a similar ability to modulate systemic therapy. Furthermore, CYP3A4-related oxidative metabolism does not appear to be a major pathway involved in the action of lenvatinib alone or coadministration with RT. There is a pressing need to incorporate our current understanding of the systemic effects of localized irradiation into future treatment strategies, and the current findings can serve as a starting point for the scientific community to explore the effects of combined lenvatinib and RT as synergistic tools in HCC treatment strategies.

## Figures and Tables

**Table 1:** Pharmacokinetic parameters of lenvatinib in rats after administration for 3 d (3 mg/kg, p.o.) with or without radiotherapy (RT, 2 Gy and 9 Gy) and ketoconazole (KTZ, 10 mg/kg, i.p., 30 min, before RT or lenvatinib).

Pharmacokinetics (PK)			Radiotherapy (RT)					CYP3A4 Modulation			
			RT_2Gy_		RT_9Gy_			RT_2Gy_		RT_9Gy_	
PK Parameters	Unit	Lenvatinib Only 3 mg/kg (*n* = 6)	RT Concurrent with Lenvatinib 3 mg/kg (*n* = 6)	RT Followed by Lenvatinib 3 mg/kg (*n* = 6)	RT Concurrent with Lenvatinib 3 mg/kg (*n* = 6)	RT Followed by Lenvatinib 3 mg/kg (*n* = 6)	KTZ Pretreatment + Lenvatinib 3 mg/kg (*n* = 6)	KTZ Pretreatment + RT Concurrent with Lenvatinib 3 mg/kg (*n* = 6)	KTZ Pretreatment + RT Followed by Lenvatinib 3 mg/kg (*n* = 6)	KTZ Pretreatment + RT Concurrent with Lenvatinib 3 mg/kg (*n* = 6)	KTZ Pretreatment + RT Followed by Lenvatinib 3 mg/kg (*n* = 6)
AUC_0-T_	min·µg/mL	87.85 ± 38.2	42.96 ± 20.7 ^a^	106.5 ± 41.8 *	44.83 ± 18.0 ^a^	75.70 ± 15.1 **	58.53 ± 11.1	35.06 ± 13.5 ^#^	42.18 ± 23.1 ^b^	58.19 ± 42.8	56.53 ± 22.6
T_max_	min	67.50 ± 55.1	152.5 ± 87.2	105.0 ± 52.8	110.0 ± 64.1	112.5 ± 79.2	123.3 ± 101	135.0 ± 86.4	90.00 ± 74.1	72.50 ± 30.6	112.5 ± 87.8
C_max_	µg/mL	0.531 ± 0.29	0.221 ± 0.1	0.561 ± 0.23	0.233 ± 0.1	0.393 ± 0.08	0.363 ± 0.13	0.183 ± 0.07	0.280 ± 0.13	0.318 ± 0.26	0.308 ± 0.11
T_1/2_	min	621.3 ± 507	304.9 ± 149	444.5 ± 469	1998 ± 2989	575.2 ± 404	222.5 ± 178	864.2 ± 879	192.4 ± 127	845.8 ± 889	888.9 ± 692

Data are expressed as the mean ± SD (*n* = 6); ^a^ *p*< 0.05 compared with the area under the concentration–time curve (AUC)_0-T_ of lenvatinib only; ^b^ *p* < 0.05 compared with the AUC_0-T_ of RT_2Gy_ followed by lenvatinib; * *p* < 0.01 compared with the AUC_0-T_ of RT_2Gy_ concurrent with lenvatinib; ** *p* < 0.01 compared with the AUC_0-T_ of RT_9Gy_ concurrent with lenvatinib; ^#^ *p* < 0.01 compared with the AUC_0-T_ of ketoconazole pretreatment before lenvatinib administration.

**Table 2:** Concentrations of lenvatinib in the heart, liver, spleen, lung, kidney, and brain of rats after administration (3 mg/kg, p.o.) with or without radiotherapy.

Organ (µg/mL)	Lenvatinib Only 3 mg/kg (*n* = 6)	RT_2Gy_		RT_9 Gy_	
		RT Concurrent with Lenvatinib 3 mg/kg (*n* = 6)	RT Followed by Lenvatinib 3 mg/kg (*n* = 6)	RT Concurrent with Lenvatinib 3 mg/kg (*n* = 6)	RT Followed by Lenvatinib 3 mg/kg (*n* = 6)
Heart	0.05 ± 0.05	0.032 ± 0.04 (↓ 36.0%)	0.074 ± 0.04 (↑ 48.0%)	0.015 ± 0.04 (↓ 70.0%)	0.078 ± 0.04 (↑ 56.0%)
Liver	0.104 ± 0.05	0.072 ± 0.06 (↓ 30.8%)	0.158 ± 0.09 (↑ 51.9%)	0.093 ± 0.05 (↓ 10.6%)	0.161 ± 0.07 (↑ 54.8%)
Spleen	0.03 ± 0.05	0 (↓ 100.0%)	0.073 ± 0.04 (↑ 143.3%)	0 (↓ 100%)	0.06 ± 0.06 (↑ 100%)
Lung	0.108 ± 0.04	0.033 ± 0.04 ** (↓ 69.4%)	0.092 ± 0.03 (↓ 14.8%)	0.042 ± 0.05 * (↓ 61.1%)	0.075 ± 0.03 (↓ 30.6%)
Kidney	0.175 ± 0.05	0.113 ± 0.03 * (↓ 35.4%)	0.23 ± 0.05 (↑ 31.4%)	0.129 ± 0.05 (↓ 26.2%)	0.223 ± 0.05 (↑ 27.4%)
Brain	0	0	0	0	0

Data are expressed as the mean ± SD (*n* = 6); * *p* < 0.05 compared with the AUC_0-T_ of lenvatinib only; ** *p* < 0.01 compared with the AUC_0-T_ of lenvatinib only; ↓ Concentration decreased when compared with the sham group; ↑ Concentration increased when compared with the sham group.

## Data Availability

The datasets used and/or analyzed are available from the corresponding author on reasonable request.
